# Reinforced Odor Representations in the Anterior Olfactory Nucleus Can Serve as Memory Traces for Conspecifics

**DOI:** 10.1523/ENEURO.0143-25.2025

**Published:** 2025-07-24

**Authors:** Christiane Linster, David Wolf, Wolfgang Kelsch

**Affiliations:** ^1^Department of Neurobiology and Behavior, Cornell University, Ithaca, New York 14850; ^2^Department of Psychiatry and Psychotherapy, University Medical Center, Johannes Gutenberg University, Mainz 55131, Germany; ^3^Department of Psychiatry and Psychotherapy, Central Institute of Mental Health, Medical Faculty Mannheim, Heidelberg University, Mannheim 68159, Germany

**Keywords:** computation, conspecific, learning, memory, olfactory, oxytocin

## Abstract

Recognition of conspecific individuals in mammals is an important skill, thought to be mediated by a distributed array of neural networks, including those processing olfactory cues. Recent data from our groups have shown that social memory can be supported by olfactory cues alone and that interactions with an individual lead to increased neural representations of that individual in the anterior olfactory nucleus, an olfactory network strongly modulated by the neuropeptide oxytocin. We here show, using a computational model, how enhanced representations in the AON can easily arise during the encoding phase, how they can be modulated by OXT, and how a dynamic memory signature in the form of enhanced oscillations in the beta range arises from the architecture of the neural networks involved. These findings have implications for our understanding how social memories are formed and retrieved and generate further hypotheses that can be tested experimentally.

## Significance Statement

Memory for conspecifics is often measured as a decrease of behavioral response compared with a novel conspecific. While behavioral responses decrease, it has been shown that neural responses to the familiar conspecific increase rather than decrease. We here use a computational model to show how increased neural responses arise from known circuitry and to suggest a mechanism underlying the decreased behavioral response.

## Introduction

**Figure 1.**
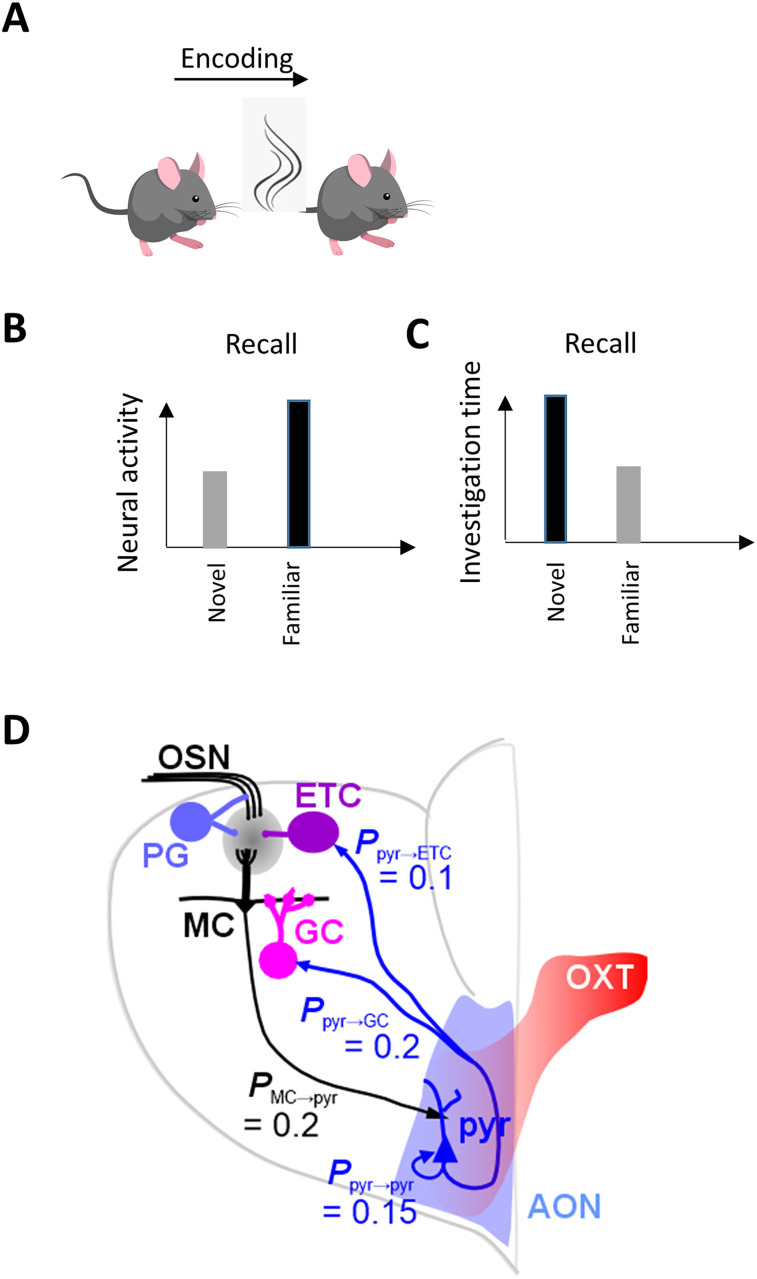
Schematic representation of experimental findings (Wolf et al., 2024). After encoding of a conspecific odor (***A***), neural activity in response to the familiar odor is increased (***B***), while investigation of that familiar odor is decreased compared with that of a novel odor not encountered (***C***). ***D***, Schematic network setup. The computational model includes sensory neurons (OSNs) projecting to OB periglomerular (PG), external tufted (ET), and mitral cells (MC). These cells are connected in a glomerular network with PGs inhibiting ETs, ETs exciting MCs, and ETs exciting each other in a small surround. This network performs normalization on the incoming odor information (Cleland and Sethupathy, 2006; Cleland et al., 2007). MCs are reciprocally connected to granule cells (GCs) with MCs exciting a random subset of 25% existing GCs and GCs inhibiting only the MC closest to it. MCs make excitatory connections with a subset of 20% randomly chosen pyramidal (pyr) in the AON network. AON pyr cells form a loose association network by connecting to each other with a 15% connectivity. Pyrs project back to OB cells with the same connectivity described before (Linster and Kelsch, 2019). Table 1 details all the parameters chosen for the present simulations.

Conspecific recognition in most mammals, and rodents in particular, relies on olfactory processing of conspecific odors. Specific neuropeptides such as oxytocin (OXT) and vasopressin have been shown to play critical roles for encoding and retrieval of conspecific odors (for review, see Walum and Young, 2018). The neural circuitry involved in these processes rely on olfactory information processing in the olfactory bulb (OB), relayed to brain areas involved in discrimination, social memory, bonding, and contextual information. Recent data has strongly implicated the anterior olfactory nucleus (AON) in the formation and expression of social memory, since depletion of OXT receptors in the AON prevented the behavioral expression of this memory (Oettl et al., 2016; Oettl and Kelsch, 2018). Computationally, recognition memory could be implemented by suppressing the encoded odor and thereby suppressing the associated behavioral response (Brennan and Kendrick, 2006; Linster and Kelsch, 2019); however, recent data suggest an alternative hypothesis based on reinforcing the encoded odor and creating stronger neural responses in the AON (Wolf et al., 2024). Neural responses in the AON of awake behaving mice to social odors showed that pyramidal cell responses to a familiar conspecific odor are enhanced after learning (Fig. 1*A*,*B*), as is the representational distance to a novel odor. Interestingly, stronger beta range dynamics were also observed specifically in response to the familiar odor. We here use our large-scale computational model to explore the circuit mechanisms underlying these new findings. We show that activity-dependent plasticity within the AON network can induce all the experimentally observed phenomena, including the emergence of odor specific beta range oscillations. Our simulations show that (1) odor encoding in the AON can be supported by the existing dense network of pyramidal cells, (2) boosting of neural activity by rheobase decrease (Oettl et al., 2016; Linster and Kelsch, 2019) via OXT inputs can modulate this encoding by increasing plasticity, and (3) familiar and novel odors can acquire differential representations reactivated by the odors via this process. The internal dynamics of pyramidal cells (Barkai and Hasselmo, 1994; McGinley and Westbrook, 2011), paired with enhanced intracortical interactions in response to odor-induced plasticity, leads to the emergence of beta oscillations, which have been observed in the olfactory networks after odor learning (Martin et al., 2004; Kay et al., 2009; Kay and Beshel, 2010; Osinski et al., 2018), but so far no specific mechanism has been pinned down. Our simulations provide a window into the mechanisms of social memory and underlying changes in neural processes. Memory for a familiar odor can be expressed behaviorally by overall shorter investigation of the familiar conspecific (Sanchez-Andrade et al., 2005; Oettl et al., 2016; Zhang et al., 2016; Meira et al., 2018; Fig. 1*C*). We suggest that faster accumulation of neural information leads to reaching a decision threshold more quickly in the case of the familiar odor, in agreement with the observed lower investigation times. After learning a familiar odor, this odor can be identified at significantly lower concentrations in the model, suggesting a decrease in approach distance during behavioral encounters.

## Materials and Methods

### Computational modeling

The model is based on a previous model by Linster and Kelsch (2019) and (Levinson et al., 2020) with added functionality in the AON network to explore the role of plasticity in this network.

#### Neurons and synapses

Our model is composed of single compartment leaky integrate-and-fire neurons, with the exception of mitral cells (MC) which are modeled as two compartments. Changes in membrane voltage *v*(*t*) over time in each compartment are described by Equation 1:

$$\tau \frac{dv}{dt}+v(t)=V^{\mathrm{ext}}\;(t),$$

where *τ* is the membrane time constant and *V*^ext^(*t*) is the voltage change resulting from external inputs (synaptic or sensory).

Each one of the voltage changes due to external inputs *V*^ext^ is a result of the synaptic strength of the connection from neuron *j* to neuron *i* (*w_ij_*) and the respective synaptic conductance in cell *i* at time *t* (*g_i_*(*t*)). *E_N,ij_* is the Nernst potential of the synaptic current and *v_i_*(*t*) is the membrane potential of the postsynaptic neuron *i*, as described in Equation 2:

$$v_{i}^{\mathrm{ext}}\;(t)=w_{ij}\;g_{i}\;(t)[E_{N,ij}-v^{i\;(t})].$$

The communication between neurons happens via discrete spikes. The spiking output *F*(*v*) of a given neuron *i* is a function of its membrane potential *v* and the minimal threshold and saturation threshold of the output function, *θ*^min^ and *θ*^max^. Where *F_i_*(*v*) = 0 if *v *≤* θ*^min^ and *F_i_*(*v*) = 1 if *v *≥ *θ*^max^ and *F*_i_(*v*) increase linearly between *θ*_min_ and *θ*_max_.

*F*_i_(*v*) defines their instantaneous firing probability and OXT modulation decreases *θ*_max_ to increase excitability. The time course of the conductance change is calculated as follows:

$$g_{i}\;(t)=g^{\max}\;\left(e^{\frac{-t}{\tau_{1}}}-e^{\frac{-t}{\tau_{2}}}\right),$$

where *g*^max^ is a constant with no unit representing the maximum conductance of a given channel and is equal to 1 (synaptic strength is scaled by the synaptic weight *w*), while *τ*_1_ and *τ*_2_ are the rising and falling times of this conductance. After firing, the spike of each spiking neuron is reset to *V*_rest_.

Local field potential data from the simulations was created by taking the average membrane potential fluctuations not including action potentials. This simulates a low-pass filter recording with a low impedance electrode.

#### Spike rate adaptation

**Figure 2.**
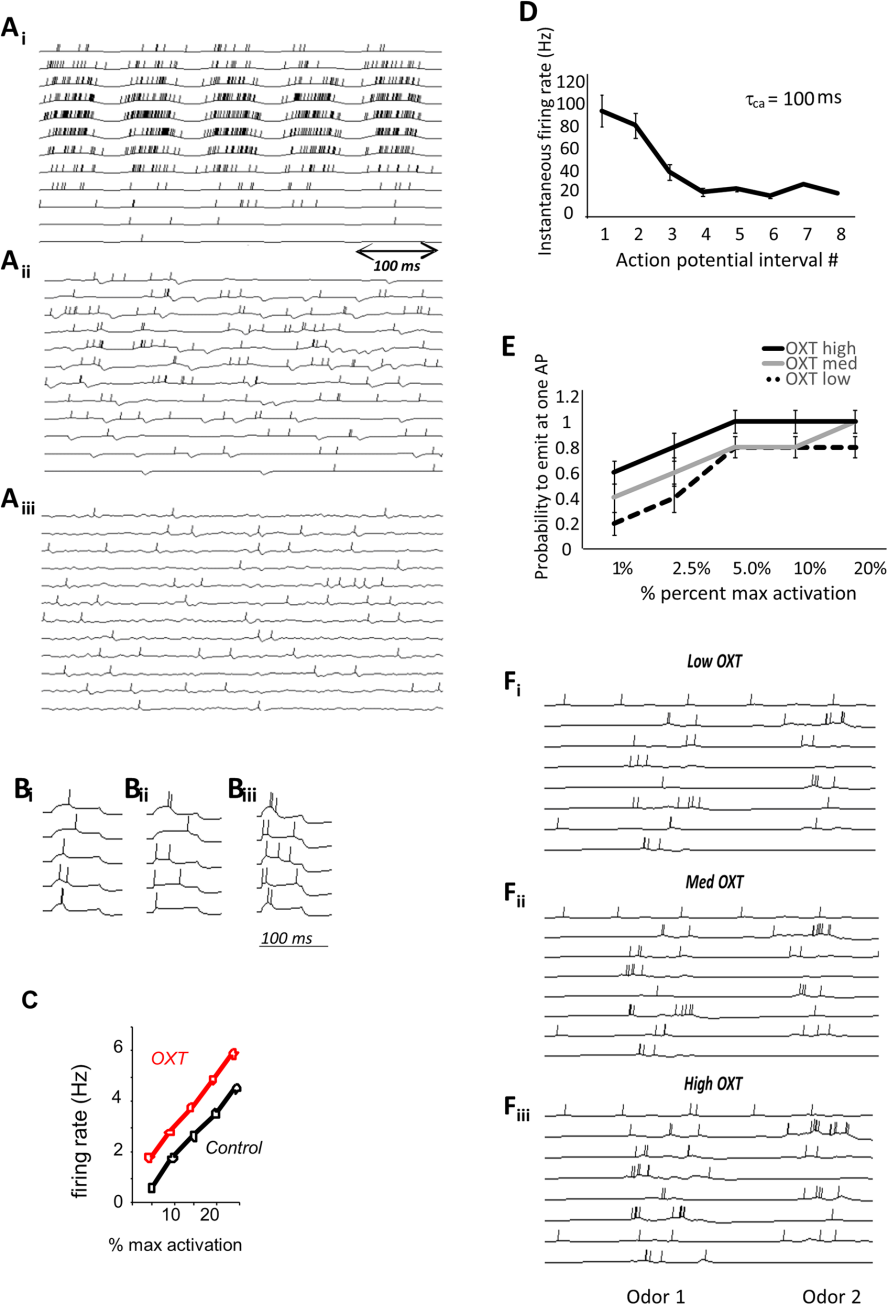
***A***, Examples of OB neural responses in the model OSNs (***Ai***), MCs (***Aii***), and GCs (***Aiii***). ***B***, Simulated responses of AON pyramidal cells to a short (100 ms) stimulation above the rheobase under low OXT (***Bi***), medium OXT (***Bii***), and high OXT (***Biii***) parameters. OXT affects response thresholds in the model with more OXT leading to lower spike thresholds (Oettl et al., 2016). ***C***, AON pyramidal cell firing rates as a function of activation currents under high OXT (OXT) and medium OXT (control) conditions. ***D***, Instantaneous firing rate in simulated AON pyramidal cells as a function of action potential interval number. These simulations served to adjust the time constant for calcium accumulation that governs the spike rate adaptation as measured in McGinley and Westbrook (2011). Ten instantiations of a pyramidal cell were run and average instantaneous frequency computed for the first 8 action potentials emitted by the cell to adjust the time constant for spike rate adaptation. The graph shows average instantaneous frequencies for the parameters chosen (tau = 100; compare with the graph shown in McGinley and Westbrook, 2011). ANOVA with frequency as dependent variable and action potential number as factor shows a significant overall effect of action potential number (*F*_(7,72)_ = 38.895; *p* < 0.001) as well as a significant negative correlation between action potential number and frequency (*R* = −0.733; *p* = 0.01). ***E***, Rheobase modulation by simulated OXT levels. The graph shows the probability to emit at least one action potential as a function of activation levels for AON pyramidal cells under the three OXT conditions. The probability to emit an action potential was calculated over 10 repeated simulations using a range of artificial activation values. ANOVA with probability as dependent variable and input and OXT levels as main effects shows a significant effect of input level (*F*_(4,135)_ = 9.952; *p* = 0.001) and OXT level (*F*_(2,135)_ = 6.404; *p* = 0.002). ***F***, Examples of neural responses of a small group of simulated AON cells to stimulation with 200 ms of two conspecific odors when OXT is low (***Fi***), medium (***Fii***), or high (***Fiii***).

Spike rate adaptation was implemented in Pyr cells as a calcium-dependent K^+^ channel which leads to a hyperpolarizing current in the cell (Barkai and Hasselmo, 1994; McGinley and Westbrook, 2011). The calcium variable was dependent on the neuron's firing and increased with each spike and slowly decreased over time using a first-order differential equation with *τ*_ca_ as time constant (see Table 1 for detailed parameters), *V*_N_ = −90 mV, and *A*^ahc^ the amplitude of the effect for the K^+^ conductance. The parameters of this equation were similar to those used for hippocampal and olfactory cortical neurons and adjusted to match data recorded in AON pyramidal cells (McGinley and Westbrook, 2011; Fig. 2*E*).

**Table 1.** Computational modeling parameters and explanations

OSN	*τ* = 1 ms; *V*_rest_ = −65 mV; *θ*^min^ = −65 mV; *θ*^max^ = −55 mV	Unless otherwise specified, neural parameters were chosen based on previous OB models to stay consistent. All synaptic weights and threshold parameters are chosen from a uniform distribution ±10% around the average given here. Reversal potentials are based on experimental results. Time constants are meant to reproduce those reported when available *θ*^max^ chosen to adjust rheobase to simulate different OXT conditions as reported in Oettl et al. (2016)
Mitral	*τ* = 5 ms; *V*_rest_ = −65 mV; *θ*^min^ = −64 mV; *θ*^max^ = −55 mV	
PG	*τ* = 2 ms; *V*_rest_ = −65 mV; *θ*^min^ = −65 mV; *θ*^max^ = −60 mV	
GC	*τ* = 4 ms; *V*_rest_ = −65 mV; *θ*^min^ = −64 mV; *θ*^max^ = −60 mV	
ET	*τ* = 2 ms; *V*_rest_ = −65 mV; *θ*^min^ = −65 mV; *θ*^max^ = −60 mV	
Pyr	*τ* = 10 ms; *V*_rest_ = −65 mV; *θ*^min^ = −62 mV; *θ*^max^ = −55 mV/−60 mV/−65 mV*	
OSN to PG	*w* = 0.0015; *E*_N_ = +70 mV; *τ*_1_ = 1 ms; *τ*_2_ = 2 ms	
OSN to Mi (apical)	*w* = 0.015; *E*_N_ = +70 mV; *τ*_1_ = 1 ms; *τ*_2_ = 2 ms	
OSN to ET (apical)	*w* = 0.0015; *E*_N_ = +70 mV; *τ*_1_ = 1 ms; *τ*_2_ = 2 ms	
PG to Mi (apical)	*w* = 0.002; *E*_N_ = −5 mV; *τ*_1_ = 2 ms; *τ*_2_ = 4 ms	
ET to Mi (apical)	*w* = 0.0015; *E*_N_ = 70 mV; *τ*_1_ = 1 ms; *τ*_2_ = 2 ms	
Mi (soma) to GC	*w*_naive_ = 0.0001 *E*_N_ = +70 mV; *τ*_1_ = 1 ms; *τ*_2_ = 2 ms; *p* = 0.25;	Chosen to reproduce reliable gamma dynamics in response to odors (Linster and Escanilla, 2019)
GC to Mi (soma)	*w* = 0.0015; *E*_N_ = −10 mV; *τ*_1_ = 2 ms; *τ*_2_ = 4 ms; local only	Chosen to be local based on McIntyre and Cleland (2016)
Mi (soma) to Pyr	*w* = 0.007; *E*_N_ = +70 mV; *τ*_1_ = 1 ms; *τ*_2_ = 2 ms; *p* = 0.20	Adapted from de Almeida et al. (2013) to match data in Poo and Isaacson (2009); Davison and Ehlers (2011)
Pyr to ET	*w* = 0.0015; *E*_N_ = +70 mV; *τ*_1_ = 1 ms; *τ*_2_ = 2 ms; *p* = 0.1	
Pyr to GC	*w* = 0.0015; *E*_N_ = +70 mV; *τ*_1_ = 1 ms; *τ*_2_ = 2 ms; *p* = 0.2	
Pyr adaptation	*A*^ahc^ = 10; *E*_N_ = −90 mV; *τ*^ahc^ = 100 ms	Adjusted to match data shown in McGinley and Westbrook (2011)

*τ*, membrane time constant; *V*_rest_, resting membrane potential; *θ*^min^, spiking threshold; *θ*^max^, saturation threshold; *w*, synaptic weight; *E*_N_, reversal potential; *τ*_1_, rise time; *τ*_2_, decay time; *A*^ahc^, afterhyperpolarization magnitude; *τ*^ahc^, calcium accumulation time constant.

* Different values are without/medium/strong OXT modulation, respectively.

#### Modulation of AON pyramidal cells by OXT

Modulation of AON pyramidal cells by OXT was modeled by adjusting pyramidal cell excitability (lowering firing threshold) to modulate the effects of OXT on the rheobase as shown in Oettl et al. (2016) (Fig. 2*D*,*F*). Pyramidal cell parameters were adjusted to reflect experimental data under control and OXT modulation conditions (Fig. 2*G*), as explored in Linster and Kelsch (2019), and we further lowered the excitability to represent the low OXT condition mimicking the knock-out. Parameters are detailed in Table 1.

#### Plasticity

In the simulations presented here, simulated exposure to an odorant induced activity-dependent plasticity of synapses between pyramidal cells in the AON network. Synaptic strengths were first calculated from the parameters given in Table 1. During simulated odor exposures, synapses between Pyrs underwent synaptic potentiation:

$$w_{ij-\mathrm{new}}=w_{ij-\mathrm{old}}+\;\alpha \;\;*\sum_{t_{1}}^{t_{2}}x_{i}(t)\;\;*\sum_{t_{1}}^{t_{2}}x_{j}(t),$$

where *w_ij_* is the synaptic strength between the presynaptic and postsynaptic Pyr, *α* is the rate of potentiation, and *x_j_* and *x_i_* are the total numbers of spikes emitted by the pre- and postsynaptic cells during the preceding sniff cycle between *t*_1_ and *t*_2_.

### Network architecture

The modeled OB network incorporates five neuron types: olfactory sensory neurons (OSN), mitral cells (MC), external tufted cells (ET), periglomerular cells (PG), and granule cells (GC). Each group is composed of 100 neurons organized in functional columns. MCs make synapses with 25% of GCs (*p*_MC-GC_ = 0.25) and GCs make inhibitory local synapses only (McIntyre and Cleland, 2016). Parameters for OB network have been extensively adjusted in previous models to mimic experimental data on odor responses and OB dynamics (cite). Here, the AON is represented by 100 pyramidal cells (Pyr). Because the connectivity between OB and AON is still poorly understood, synapses between mitral and pyramidal cells were created randomly with each mitral cell projecting to any pyramidal cell with an equal probability of *p*_MC-Pyr_ = 0.2 as shown in Figure 2*A*. The number and strength of these connections was similar to those we used for piriform cortex models (de Almeida et al., 2013, 2015, 2016), in the absence of specific data on AON responses. Neural and synaptic data was adjusted in our previous AON model to replicate AON odor responses and spontaneous activity to the extent possible given the sparseness of available data. In the simulations presented here, the focus was on learning of social odors the AON, not the role of feedback to the OB; as a consequence, AON projections to the OB were kept very weak here in order to minimize their interference. Intra-AON connections between pyramidal cells were modeled with low initial synaptic weights and connectivity *p*_pyr-pyr_ = 0.15; these were subject to activity-dependent plasticity. The connectivity was chosen to allow for context addressable odor memory (enough connections) but prevent run–away synaptic activity (too many connections).

### Simulated odor exposure and learning of a familiar odor

We simulated odor exposures and learning of the familiar in the following manner. Two odors, representing the familiar and novel odors, were chosen such that they each activated 20–25% or OSNs with a Gaussian distribution of activities (maximal activity = 1.0 for full concentration) and a small degree of overlap. To test the effect of learning, we first simulated exposure to no odor, a familiar odor, and a novel odor for a 0.5 s simulation time to measure baseline neural activity and odor-evoked neural activity in the model. We then presented the model with the familiar odor for a 5 s interval during which plasticity between neurons in the AON was possible. Five seconds are within the time of what has been reported for animals investigating conspecifics (Oettl et al., 2016) and sufficient here to produce significant changes in synaptic strength in the model. Note that learning rates and learning time interact in modeling, and similar results can be obtained with higher rates and shorter learning time or lower rates and longer learning times. As noted above, degree of synaptic change was calculated from pre- and postsynaptic activity in the preceding sniff cycle, and weights were updated in between sniff cycles. After the presentation of the familiar odor with plasticity active, we presented the familiar odor, novel odor, and no odor again for 0.5 s each to assess the effect of learning on odor representations.

### Implementation

All simulations were implemented using the C programming language in a Linux environment (Ubuntu 14.04 LTS x64) on an Intel desktop computer, with Euler integration method for the differential equations with a time step of 1 ms (for short odor presentations up to 5 s) or 100 ms (for longer presentations). For each instantiation of the model, all parameters were chosen from a uniform random distribution ±10% around the mean values detailed in Table 1 to prevent results being based on specific ratios of parameters. All odor exposure were simulated at least five times (detailed numbers are in the figure legends) with a new initiation of the model for each run.

### Code accessibility

The code/software described in the paper is freely available online at https://github.com/clinster/Linsteretal2025.

### Analysis

To compare neural activities in response to odors pre- and postlearning, we used compared pyramidal cell firing rates in response to at least five (numbers are specified in each figure legend) simulations using a new model setup with new seed for randomization. Euclidean distances between population responses were computed by calculating average rates during the time interval of interest for all AON pyramidal cells and using these rates to create *N*-dimensional vectors, with *N* the number of neurons. Details of each analysis can be found in the figure legends. All analyses were performed using SPSS.

## Results

### Neural activity in the model

Neural activity in our large-scale OB model has been adjusted to reflect known characteristics of odor responses (de Almeida et al., 2013, 2015, 2016; Mandairon et al., 2014; Linster and Escanilla, 2019; Linster and Kelsch, 2019; Levinson et al., 2020). Figure 2*B* shows simulated neural activity in the OB in response to an odorant. OSNs are activated periodically in response to simulated breathing (*Bi*), with a subset of OSNs responding to an odor with a range of firing rates. Mitral cells in the olfactory bulb can be excited or inhibited in response to odorants (*Bii*) and are similarly driven by respiration. Granule cells, depicted in *Biii*, show strong subthreshold oscillations in the gamma range with action potentials riding on top. AON pyramidal neurons express OXT receptors (OXTR) and are densely targeted by axonal projections of OXT neurons (Knobloch et al., 2012). Figure 2*C* shows responses of AON pyramidal cells to brief current injections (100 ms) while simulating NO (*Ci*), medium (*Cii*), and high (*Ciii*) OXT levels. In *D*, we show how simulated OXT increases firing rate in response to current activation (compare with data shown in Oettl et al., 2016). Spike rate adaptation in pyramidal cells was adjusted to match that published in Barkai and Hasselmo (1994); McGinley and Westbrook (2011); the graph in Figure 2*E* shows the instantaneous firing rate as a function of action potential number in pyramidal cells. Figure 2*F* shows how pyramidal cells respond to odorants under NO OXT (simulating the knock-out situation), medium OXT [simulating the natural (control) state], and high OXT (simulating the optogenetic activation state). Figure 2*G* shows responses to AON pyramidal cells to two simulated odors under medium OXT conditions (these are simulating the “natural” OXT levels). In the model, odor responses to a simulated familiar odorant increase with OXT levels, as predicted by simulated current input in Figure 2*D*.

### Effects of learning on neural responses in the AON

**Figure 3.**
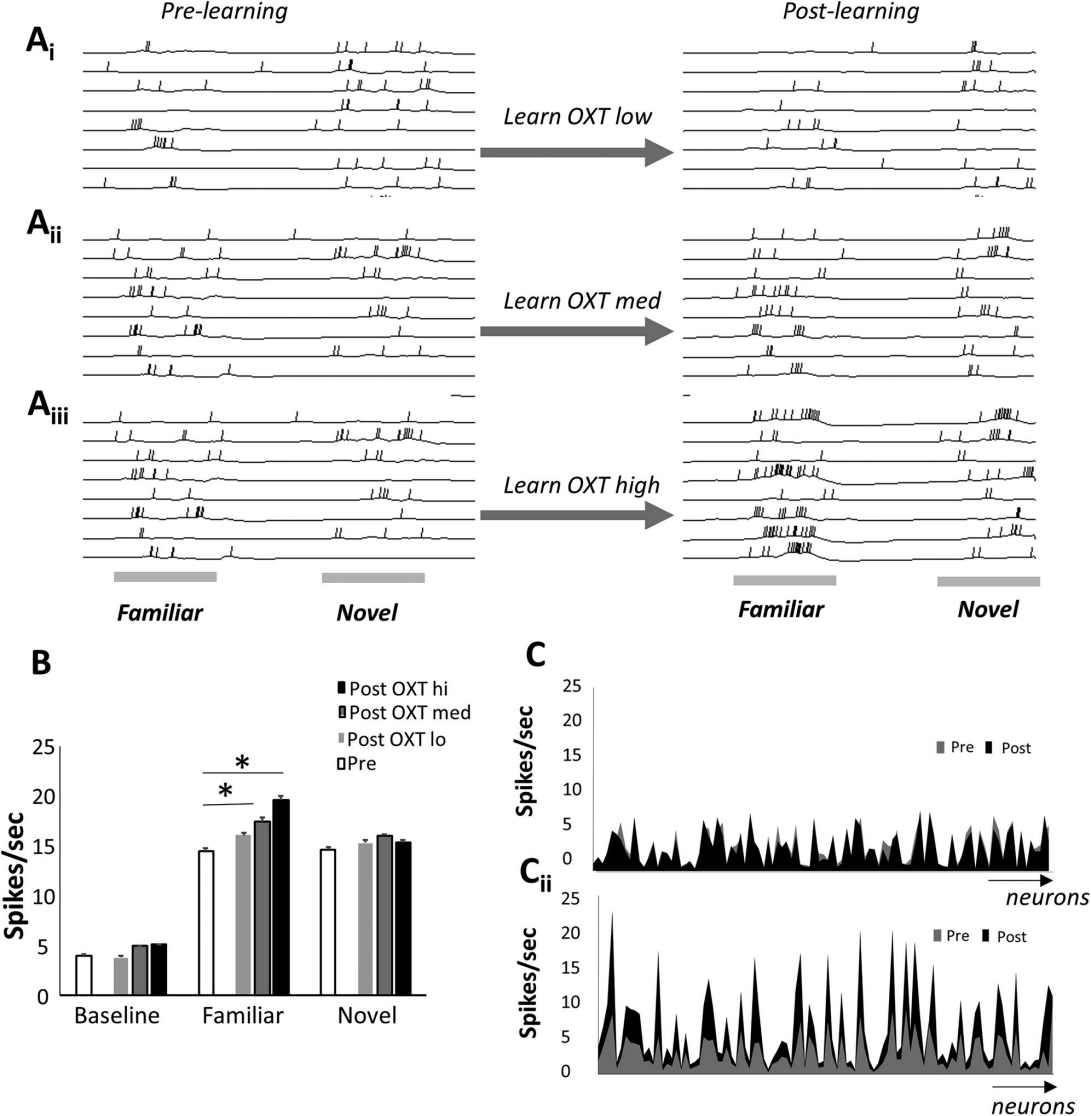
Learning increases odor responses to the learned odor. ***A***, Example of neural activity in the model pyramidal cells to the familiar and novel odor before and after learning the familiar odor with low OXT (***Ai***), medium OXT (***Aii***), and high OXT (***Aiii***). ***B***, Average firing rates in model pyramidal cells (spikes per second) during baseline activity and odor-evoked activity before and after learning of the familiar odor under three OXT conditions. To obtain these data, 10 instantiations of the model were run through the following simulations: baseline activity, odor F, and odor N each for 0.5 s with medium OXT. Odor F was then presented for 5 s with plasticity turned on (learning rate > 0.0). After that, baseline, odor F, and odor N were presented for 0.5 s again and the average number of spikes were recorded. Each of set of 10 simulations was run three times with plasticity under low, medium, and high OXT levels. The number of spikes in response to each odor was compared pre- and postlearning using paired *t* tests with alpha < 0.01 to account for multiple comparisons. Responses to the familiar odor F were significantly higher after learning under medium and high OXT as compared with prelearning (*p* < 0.001). Responses to odor N were not changed by learning of odor F (*p* > 0.2 in all comparisons). ***C***, Example average activity levels of pyramidal cells on response to novel (***Ci***) and familiar (***Cii***) odors before and after learning. Note that neurons with higher firing rate responses are more likely to find their responses enhanced. Neurons from 1 to 100 are shown on the *x*-axis with average firing rates on the *y*-axis.

We then simulated encoding and recall of individuals as performed in the experimental work our model is based on Wolf et al. (2024). We first present 0.5 s of simulation time of a familiar (F) and novel (N) odor under medium OXT levels to record prelearning responses to these two odors (Fig. 3*A*, pre-learning). To compare our simulations to published data, we then presented the model with odor F for 5 s simulation time while plasticity in the AON was allowed [under low (corresponding to OXTR knock-out in the AON), medium (natural OXT levels), or high (additional optogenetic stimulation of OXT)] and subsequently recorded responses to F and N again under medium OXT for 0.5 s again (Fig. 3*A*, post-learning). Activity-induced plasticity between Pyrs in the AON resulted in changes in neural activity specific to the learned odor F. When OXT is absent during learning of odor F, simulating learning in the OXTR knock-out mouse, odor-induced activity is reduced, and synaptic weights are not dramatically increased, resulting in no change in response to odors N and F. With medium OXT, simulating the natural situation, weights are increased and neural activity in response to the familiar odor increases (postlearning). With extra OXT (high), simulating optogenetic activation of OXT, subsequent responses to the familiar odor are increased even more. Average pyramidal cell firing rates (all neurons, not only those activated by the odor stimulation) increased significantly above the prelearning odor responses only for the familiar odor (F) and only for learning under medium or high OXT conditions (Fig. 3*B*). Figure 3*C* shows examples of average neural activities across the AON prelearning (gray line) and postlearning (black line) of the familiar odor for odor N (*Ci*) and F (*Cii*) under medium OXT conditions. Increases in spike rate were specific to the learned odor F (as observed experimentally; Wolf et al., 2024).

### Increased detection and discrimination of familiar odor after learning

**Figure 4.**
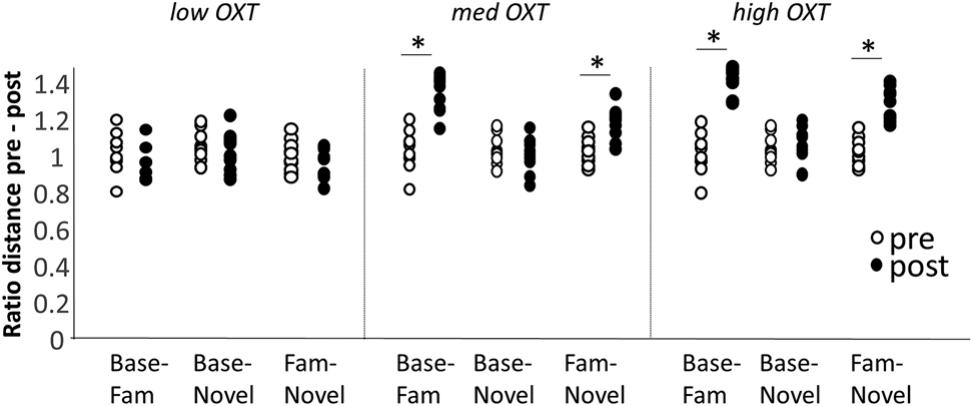
Euclidean distances between odors and baseline as well as learned odors and novel odors before and after learning. The graph shows distributions of distances between baseline-familiar, baseline-novel, and familiar-novel neural activities pre (white dots) and post (black dots) learning. Simulations were run as described in Figure 3*B*. All data are normalized by the average distance before learning to facilitate comparison. Pre- and postlearning distances were compared using repeated measures in SPSS. Low OXT: baseline-familiar *F*_(1,9)_ = 5.112; *p* = 0.054; baseline-novel *F*_(1,9)_ = 0.002; *p* = 0.961; familiar-novel: *F*_(1,9)_ = 2.212; *p* = 0.175. Medium OXT: baseline-familiar *F*_(1,9)_ = 38.242; *p* < 0.001; baseline-novel *F*_(1,9)_ = 0.155; *p* = 0.704; familiar-novel: *F*_(1,9)_ = 1.611, *p* = 0.236. High OXT: baseline-familiar *F*_(1,9)_ = 89.072; *p* < 0.001; baseline-novel *F*_(1,9)_ = 1.611, *p* = 0.236; familiar-novel: *F*_(1,9)_ = 49.211; *p* < 0.001.

Increases in firing rate per se do not necessarily signal better detection or discrimination of the learned odor. We therefore calculated the average Euclidean distances between the population vectors between spontaneous activity and odors N and F as well as between N and F, comparable with those calculated from neural recordings (Wolf et al., 2024). The distance of the activity evoked by odor N to baseline did not increase much after learning under all three conditions (Fig. 4, Base-Novel). From a computational viewpoint, this means that learning of odor F does not increase detection of N with respect to baseline. In contrast, the population distance between the learned odor F and baseline (Fig. 4, Base-Novel) increased with learning under medium and high OXT, indicating increased detection of the familiar odor after learning. Most importantly, the distance between the population activity vectors to odors N and F (Fig. 4, Fam-Novel) increased with learning under medium and high OXT modulation. These results show that learning the familiar odor increases the model's ability to detect the familiar odor and to discriminate the familiar from novel odors (Fig. 4). These results are in agreement with neural recordings from mice in our published data (Wolf et al., 2024).

### Altered neural dynamics in response to familiar odor after learning

**Figure 5.**
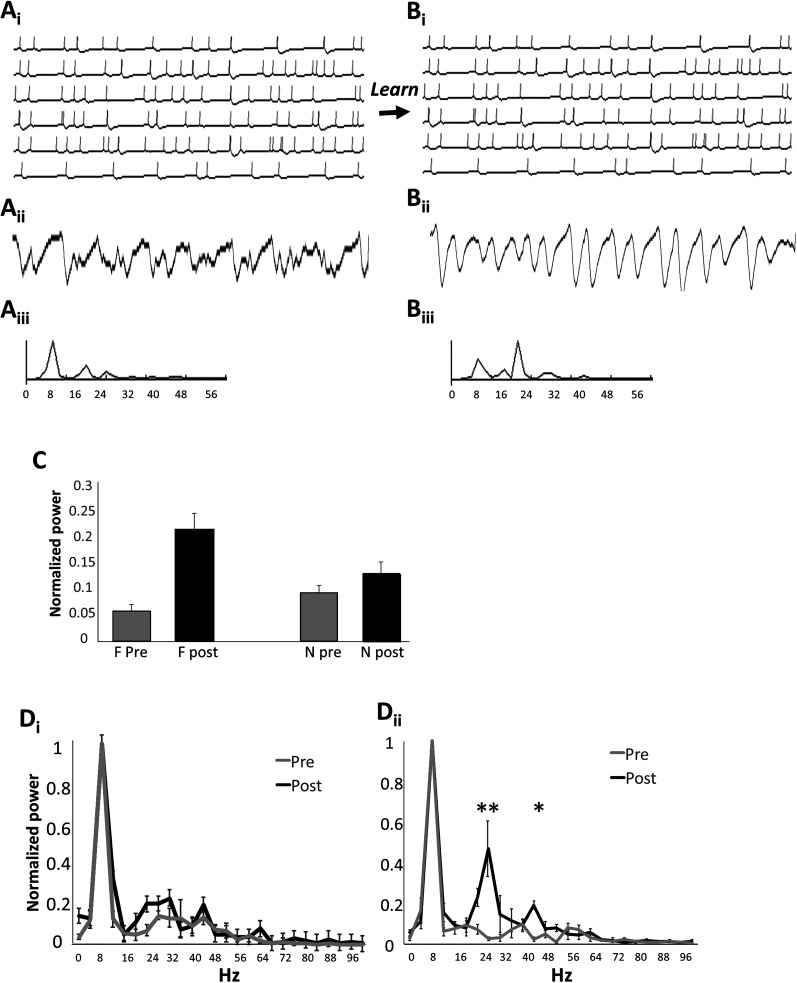
Beta range dynamics in response to learning in the pyramidal cell network. ***A***, Familiar odor prelearning and ***B***, familiar odor postlearning. Example pyramidal cell activity in response to the familiar odor before learning (***Ai***) and after learning (***Bi***), with accompanying LFP traces (***Aii*** and ***Bii***), simulated respiration (***Aiii*** and ***Biii***) and power spectrum (***Aiv*** and ***Biv***). ***C***, Power in the beta range (normalized to peak power) pre- and postlearning of odor F. Simulations were run by first presenting odors F and N for 0.5 s (pre), then presenting odor F for 5 s with plasticity on followed by a second presentation of odors F and N for 0.5 s (post). Power spectra were calculated from the simulated LFP during pre- and postlearning odor stimulations. Power was normalized to the highest peak (8 Hz) and power in the beta range was averaged (12–30 Hz). Simulations were run four times with new network instantiations. Analysis of variance with power as dependent variable and odor (F/N) and state (pre/post) as factors showed a significant effect of state (*F*_(1,12)_ = 20.769; *p* = 0.001) as well as a significant interaction between odor and state (*F*_(1,12)_ = 7.882; *p* = 0.016). Further post hoc comparisons (Tukey HD) showed a significant difference between odors postlearning (*p* = 0.048) but not prelearning (*p* = 0.117). A significant effect or learning was observed in the familiar (*p* = 0.004) but not the novel odor (*p* = 0.201). ***D***, Average power spectrum of the AON network pre- and postlearning of the familiar odor in response to the novel (***Di***) and familiar (***Dii***) odor. Simulations were those described above for ***C***. Analysis of variance with power as the dependent variable and frequency and state (pre/post) as factors showed a significant effect of frequency (*F*_(25,156)_ = 56.932; *p* < 0.001) and state (*F*_(1,156)_ = 9.061; *p* = 0.003) but no interaction (*F*_(25,156)_ = 1.171; *p* = 0.275) for the novel odor (***Dii***). In contrast, frequency, state, and their interaction were significant for the familiar odor (*F*_(25,156)_ = 93.547; *p* < 0.001; *F*_(1,156)_ = 93.547; *p* < 0.001; *F*_(25,156)_ = 7.789; *p* < 0.001). Post hoc comparisons showed that after learning, the power in response to the familiar odor is significantly higher in the beta range (*p* = 0.018 at 24 Hz, *p* = 0.001 at 28 Hz) and low gamma range (*p* ≤ 0.001 at 44 Hz).

Spike rate adaptation leads to neural dynamics of individual neurons in response to stimuli that are governed by the time constant of the spike rate adaptation. Figure 5*A* shows activity in a network of pyramidal cells with low baseline synaptic weights (*Ai*), the simulated LFP trace (average voltage across pyramidal cells in the model; *Aii*), the simulated respiration trace (*Aiii*) the corresponding power spectrum (*Aiv*) showing peaks in the theta range (∼8 Hz, respiration), and low peaks in the beta range (∼20 Hz). After learning of odor F, pyramidal neurons are connected with strong synaptic weights in a dense network and synchronization occurs. As a consequence, the frequency of the emerging population oscillation is governed by the time constant of spike rate adaptation, which corresponds roughly to the period of beta range oscillations. Figure 5*B* shows examples of individual neural activity, LFP and power spectrum after learning of odor F. This change happens as a result of synaptic plasticity during learning the familiar odor and stronger coupling. A strong peak in the beta range can be measured in the power spectrum after learning (Fig. 5*Biv*). Figure 5*C* summarized the findings across different network instantiations: the power at the peak frequency increases significantly after learning (Fig. 5*C*) and the peak frequency changes from theta range to beta range (Fig. 5*C*). Interestingly, the emergence of beta range oscillations is specific to stimulation with the learned odor as shown in Figure 5*D*. In these graphs, power is normalized to the theta peak to highlight the difference in the beta peak better. A significant difference in power compared with before learning can only be seen in response to F (Fig. 5*Dii*) but not N (Fig. 5*Di*).

### Faster accumulation of information about learned odors in the model

**Figure 6.**
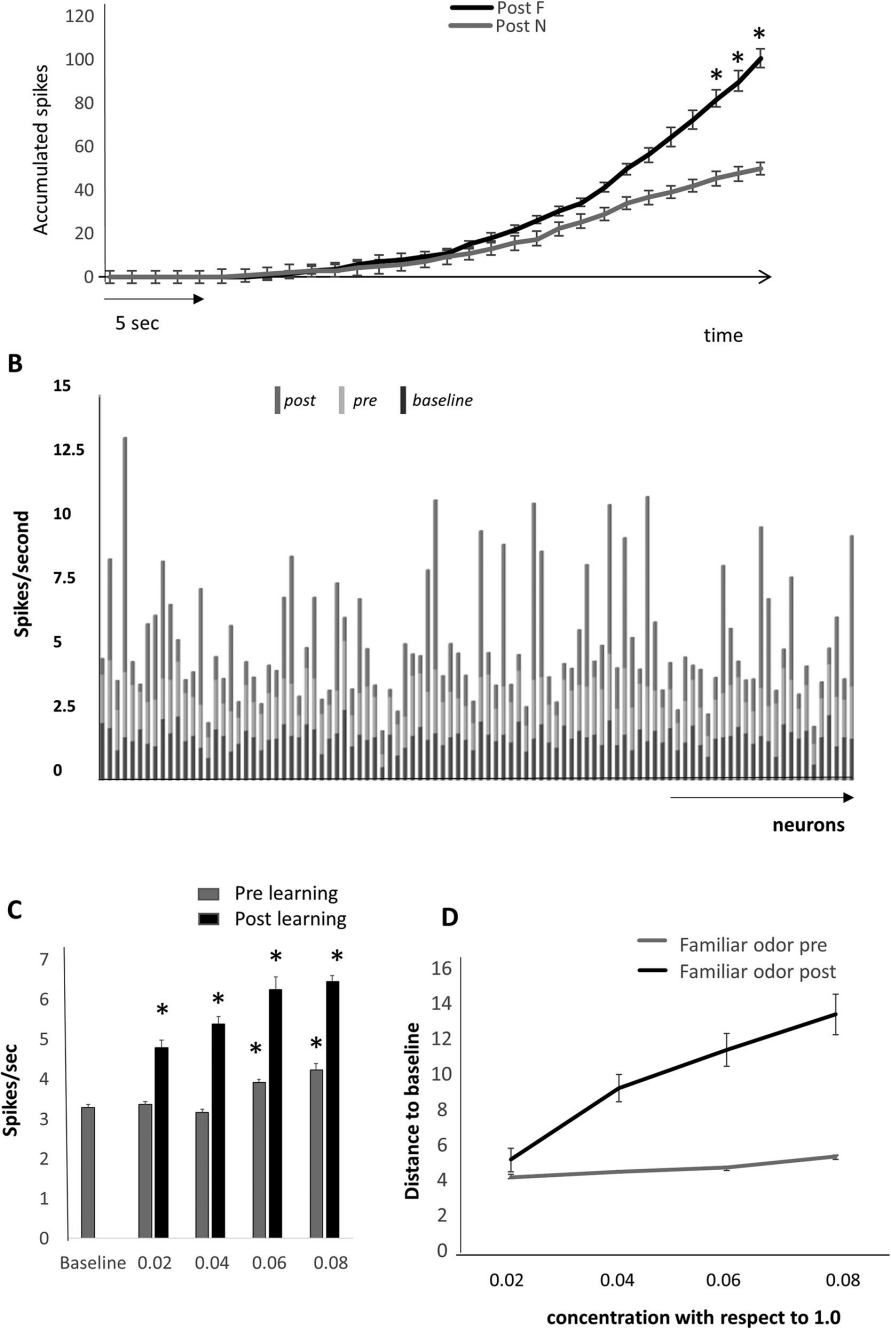
Effects of learning on neural representations. ***A***, Accumulation of action potentials in response to a familiar and a novel odor shows that a significant difference is reached ∼25–30 s simulation time. We ran five iterations of a network that first learned during 5 s of presentations of odor F (5 s presentation) and then tested the network responses to a 60 s presentation of each odor without plasticity. We here show and analyze responses to the first 30 s of these presentations. Over the course of 30 s, the number of spikes accumulated is significantly different in responses to odors F and N (*F*_(1,210)_ = 28.366; *p* < 0.001) with a significant interaction between the presentation step and the odor (*F*_(1,210)_ = 2.080; *p* = 0.002). Post hoc tests (Tukey HD) then shows a significant difference between the number of accumulated spikes after 27 s of presentation time (*p* = 0.049, 0.032 and 0.018 for 28, 29, and 30 s). ***B***, Responses to below threshold odor concentrations. The graph shows average activity in the network of pyramidal cells for a below threshold odor before (0.02 of max activation; pre) and after (post) learning of that odor as well as the baseline activity for comparison. ***C***, Average numbers of action potentials over 10 runs of the model at different low odor concentrations (concentrations are given as ratio of the concentration used for learning) before (pre) and after (post) learning the odor. There is an overall effect of concentration for both conditions (*F*_(1,41)_ = 7.428; *p* < 0.001 and *F*_(1,46)_ = 22.299; *p* < 0.001) with all concentrations significantly different from baseline after learning (*p* ≤ 0.02 in all cases using Tukey HD). Before learning, only the two higher concentrations were significantly different from baseline (*p* < 0.001 in both cases). ***D***, Average Euclidean distance to baseline activity for low concentration familiar odors before and after learning of the familiar odor. Distances were computed from data shown in ***C***.

Our modeling results suggest that faster accumulation of information in response to the familiar leads to faster recognition of a familiar odor. This putative process is illustrated in Figure 6*A*. An odor (F or N) is presented to the model and action potentials in AON cells are accumulated (running sum). In the example shown in the graph, after encoding of the familiar odor, spikes accumulate significantly faster in response to the familiar than the novel odor; after ∼25–30 s of simulation time, the number of accumulated spikes is significantly higher in response to the familiar odor as compared with a novel odor and the neural population distance between familiar and novel odor representations reaches a threshold that could drive the decision to stop investigating the familiar odor. This result is purely a proof of concept and the simulation time shown here is relative.

### Better recognition of low concentration familiar odors in the model

As shown in Figure 4, the population distance between baseline activity and activity in response to odor F is increased in response to learning. A practical consequence of enhancing familiar odor representations in the AON is that a significant difference between spontaneous and odor-evoked information is present at much lower odor concentrations (Fig. 6*B*,*C*). After learning, information about the odor is stored in the AON network and can be retrieved in response to very low concentration input from the OSNs and OB, as suggested also from experimental data in piriform cortex (Bolding and Franks, 2017; Nagappan and Franks, 2021). Figure 6*B* shows average neural activity with the AON network in response to baseline and a very low concentration odor before and after learning. After learning, the low inputs from the OB are significantly amplified by the strengthened intrinsic synapses in the AON, and the odor can easily be detected as compared with baseline. Figure 6*D* shows the average Euclidean distance from baseline at a range of increasing concentration. These data show that in the model, low concentration odors can be detected and discriminated at lower concentrations after learning.

## Discussion

Using a large-scale computational model of the olfactory system, we show that synaptic plasticity between AON pyramidal cells can account for a number of experimental observations in social odor learning: (1) enhanced neural response to a familiar odor in an olfactory cortex, (2) increased population distances between baseline and familiar odor responses, (3) emergence of beta range oscillations in the network in response to a familiar odor, and (4) modulation of these effects by OXT. We show that known cellular effects of OXT in single cells in the AON can account for the modulation by OXT of learning induced neural activity observed experimentally. We propose that faster accumulation of information—due to higher activity in response to the learned odor—leads to a reduced need for investigation. After learning, the measured difference between familiar and novel odors reaches a discrimination threshold faster such that less investigation time is needed. The observation that after learning the familiar odor can be detected at lower concentrations by model neurons could explain why investigation of a familiar odor is done with fewer close approaches than that of a novel odor.

Habituation to a familiar odor is one well-used paradigm to probe olfactory learning and memory (for review, see Wilson and Linster, 2008). In contrast to associative learning, in which an odor is associated with a reward and the behavioral response to that odor increases, habituation learning leads to a reduced response to that odor [see Cleland, Morse et al. (2002)for task comparisons]. At the neural level, these two behavioral responses have been ascribed to quite different neural mechanisms: while habituation learning is thought to be mediated by a reduction in neural activity to the learned odor, associative learning is thought to be mediated by increased responses in associative networks (Kadohisa and Wilson, 2006; Chaudhury et al., 2010; de Almeida et al., 2013; Zhang et al., 2024). Enhanced neural responses and enhanced differences between the learned familiar odor and the not learned novel odor correlate with reduced investigation times of the familiar odor during recognition trials (Sanchez-Andrade et al., 2005; Oettl et al., 2016; Zhang et al., 2016; Meira et al., 2018). Typically, after encoding the odor of a conspecific, mice will investigate a novel conspecific for ∼40–70 s and the familiar conspecific significantly less (15–30 s depending on the experimental setup). This has been assumed to be due to decreased neural activity in response to the familiar (Brennan and Kendrick, 2006; Shea et al., 2008; Chaudhury et al., 2010; Linster and Kelsch, 2019); however, our electrophysiological results show enhanced neural responses to the familiar odor, as confirmed by the present simulation results (Wolf et al., 2024). Our modeling results suggest that faster accumulation of information in response to the familiar odor can be a mechanism by which recognition can happen faster and with less time spent investigating.

Beta range dynamics have been established as a marker for olfactory learning (Martin et al., 2006; Kay and Beshel, 2010; Kay, 2014; Martin and Ravel, 2014). For example, as rats learned a go–no go odor discrimination task, beta range oscillations in the olfactory system emerged as the rats reached criterion on this task (Martin et al., 2006; Martin and Ravel, 2014). While this phenomenon is relatively well established behaviorally, the detailed mechanism is not known. Our modeling approach suggests a rather parsimonious explanation: as synaptic coupling in olfactory cortex increases during learning, pyramidal cells become more synchronized. Individual neurons’ internal dynamics lead to collective dynamics in a strongly coupled network; hence, beta range dynamics emerge due to individual neurons spike rate adaptation dynamics. Strong oscillations at this frequency range can be a signature of a learned odor, as suggested by our experimental data (Wolf et al., 2024).

In summary, we suggest that a simple plasticity mechanism can underlie behavioral and neural observations during social memory recognition behavior. Modeling OXT in the AON manipulates our modeling results in the same direction than experimental data, lending further support to our hypothesis.

## Synthesis

Reviewing Editor: James Howard, Brandeis University Decisions are customarily a result of the Reviewing Editor and the peer reviewers coming together and discussing their recommendations until a consensus is reached. When revisions are invited, a fact-based synthesis statement explaining their decision and outlining what is needed to prepare a revision will be listed below. The following reviewer(s) agreed to reveal their identity: Nancy Padilla-Coreano, Zachary Johnson.

This paper presents a computational model of oxytocinergic modulation in the anterior olfactory nucleus (AON). Inspired by previous findings regarding AON neural dynamics during familiar vs novel social odor presentations, the model simulates how varying levels of oxytocin (OXT) may influence olfactory learning and social behavior. The model simulates learning, which results in changes in synaptic weights and firing rate of AON pyramidal neurons in an oxytocin-dependent manner. The simulations also show that after learning, familiar odors result in beta-range neural oscillations. This model accounts for previously observed findings related to odor learning, such as increased beta oscillations and enhanced neural responses accompanied by reduced social investigation, and provides a novel tool for simulating behavioral paradigms and generating hypothesis. Overall, this is a timely and potentially impactful contribution to an understudied aspect of social behavior, sensory processing, and neuromodulation - how early sensory circuits might encode familiarity. The integration of computational and empirical work is a valuable and often underutilized approach in systems neuroscience. However, currently, there is no characterization of the sensory conditions that lead to plasticity and the temporal dynamics of this plasticity. This lack of characterization of the model limits its impact. Furthermore, the manuscript methods and results sections are missing information about the model and statistical comparisons that currently limits the potential impact of this research and make it hard to evaluate the rigor. Specific comments and suggestions are as follows:

Major:

1. Additional details are needed to provide clarity on how the model simulates AON plasticity and ultimately familiarization with odors. For example, for the model to learn, how much odor exposure is needed? There are no details on how many times the odor was presented to the model and how long each presentation lasted during the learning session. The authors should report this information and their rationale for picking the specific learning paradigm they selected. This information is essential for future behavioral biological experiments to benefit from this model.

2. The authors have selected parameters for the model that are mostly plausible, and in some cases directly supported by prior studies. However, it is not always clear which parameters are empirically grounded versus assumed. A table or supplemental section that explicitly categorizes parameters as either empirically supported (with citations) or based on inference would provide clarity on this issue. For parameters in the latter category, the authors should assess how sensitive the model's conclusions are to modest variations. For instance, the assumption that mitral cells (MCs) synapse with 25% of granule cells (GCs) could be tested by varying that proportion within a reasonable range (e.g., 15-35%) to determine whether the main findings are robust.

3. The authors are in the unique position to be able to model what type of temporal dynamics of odor presentation are needed to evoke plasticity, and how long this plasticity lasts. However, there is no characterization of the behavioral conditions (e.g., number of social interactions or odor interactions, duration, inter-trial interval (ITI), etc.) that would lead to this plasticity and how long it's expected to last.

4. The manuscript describes the "low" OXT condition as modeling an OXTR knockout mouse. However, a true knockout would lack oxytocin receptor signaling entirely. If this condition retains any level of OXTR activation, it may be more appropriate to describe it as a partial loss-of-function model. Clarifying this point in both the methods and discussion will help prevent misinterpretation.

5. The Methods section does not include sufficient explanation of how oxytocin level is modeled and how it impacts the model parameters exactly. Is every pyramidal neuron equally modulated by oxytocin or only a subset?

6. The Methods section does not include any information about the learning paradigm. How many odor presentations did the model receive and what is the duration of each odor presentation and ITI between odor presentations.

7. In the data shown in Figure 6A, is the odor present for the 30 seconds shown? If so, why was this chosen? This approach doesn't model how social interactions occur where there are multiple sniffing bouts across time.

8. The data in Figure 6 does not show behavior, as the model in this manuscript does not model behavioral output. However, the authors present the data with strong statements of behavioral implications in their results. This is not appropriate for the Results section. The authors should explain the analysis in Figure 6 in the Results section. Speculations of how results in figure 6 relate to behavioral observations in the literature should be in the Discussion.

9. No statistical testing information was given for all results/figures. For every figure in the manuscript, it is unclear what samples were used for the mean and error bars calculations and what statistical comparison was made. Each figure legend should have stand-alone statistical details and sample size. The Methods section indicates that "different odors and different conditions were compared using t-tests", but does not specify if its paired vs unpaired, 1 vs 2 tails and if multiple comparisons were accounted for.

10. The model predicts no differential response to odors N and F under the low OXT condition. Are OXTR knockout mice reported to have general olfactory deficits, or are impairments specific to social odors? Incorporating this point into the discussion-and citing relevant behavioral studies-would enhance interpretability.

11. OXTRs are also expressed in the olfactory bulb (OB), where they modulate early odor processing. Does the model include OB OXT/OXTR dynamics, or is it limited to the AON? If the latter, it would be helpful to acknowledge this as a limitation and briefly discuss potential interactions between OB and AON responses under varying OXT levels.

12. The manuscript states: "The code/software described in the paper is freely available online at xxx." The authors must specify where their code and software is available.

Minor:

1. Figure 1A: The schematic could more clearly reflect the olfactory context of the model. At present, it resembles a generic social interaction between two cartoon mice. The authors should consider incorporating odor-related cues (e.g., odor plumes, sniffing behavior) to emphasize the sensory modality being studied.

2. The network diagram in Figure 2A is too small making it hard to read, and seems to have inconsistent probabilities compared to those reported in the results. This network diagram is necessary but is missing a legend to highlight all the acronyms and since the main changes with learning are the synaptic weights, this should be depicted in the network, possibly with a clear legend indicating line thickness as synaptic weight. Ideally two versions of the network are side by side, one showing the network pre-learning and the other post-learning. Additionally, the networks presented in Figure 2B and Figure 7C seem identical - please clarify whether this is the case and avoid duplicating unless it is absolutely necessary.

3. Figure 3C: The fonts appear distorted or warped. Please correct this to improve legibility.

4. Figure 5: The resolution in certain panels is low or appears stretched. Improving image quality and ensuring uniform resolution across panels will improve figure readability.

5. What is the significance of the dash line in Figure 6A? This should be specified in the figure e legend.

6. For figure 6B, the way the data are plotted with connected lines makes it look like you are plotting the average firing rate over time. It took some time to realize that the authors are plotting individual neurons connected by the lines. This is a confusing way to represent this data - please consider a plot that does not involve connected lines.

7. There seems to be variability across pyramidal neurons in showing increased firing rate in 6B. Is that explained by oxytocin sensitivity or connectivity differences? Given the lack of methodological details it's unclear whether this could be the case.

8. The color scheme in Figure 7A is inverted compared to the rest of the figure and 7B needs a legend.

9. In several cortical regions, OXTRs are expressed most abundantly on interneurons, an interesting contrast to pyramidal neuronal expression here. The model also seems to assume uniform OXTR expression across all AON pyramidal cells. Is this supported by molecular data? If not, this assumption should be noted and discussed briefly.

10. The methods mention 50 simulations with different randomization seeds. What was randomized in the initiation of the model each time?

11. The methods need to explain how LFP and respiration were simulated and included in the model.

12. How were the probabilities of connections between regions chosen for this model?

13. The terminology used for the two odors is confusing. The familiar odor is called familiar even during the first presentation. This gives the impression that there was already exposure to this "Familiar odor" before the learning takes place. Then the novel odor is presumably presented twice, so it's no longer novel in second presentation. The reviewers suggest using odor A and odor B labels, then referring to odor A as the familiar odor only after the learning session.

14. "MC" is introduced before the full term "mitral cell" is defined.

15. The sentence beginning with "Neural responses in the AON of awake..." contains repeated uses of "response" and would benefit from rewording.

16. "Known plasticity in this layer" refers to an unspecified layer-please clarify.

17. Oxytocin is known to be released en passant and to diffuse via volume transmission. The manuscript would be strengthened by acknowledging these nonsynaptic mechanisms of action.

18. In the sentence "compare to Oettl, Ravi et al. 2016," it would be helpful if the authors briefly summarized how their model's predictions relate to that study, rather than leaving the comparison implicit.

19. In the statement "Odor responses to a same odorant increase with OXT levels, as predicted by simulated current input in Figure 2D," it is unclear whether this pattern holds for all odors or is specific to social cues. Please clarify and, if possible, compare with empirical data.

20. Typographical Error: In the Figure 2 legend, "exiting" should be corrected to "exciting."
